# Identification of heart failure hospitalization from NHS Digital data: comparison with expert adjudication

**DOI:** 10.1002/ehf2.14669

**Published:** 2024-01-17

**Authors:** Fardad Soltani, Joshua Bradley, Antonio Bonandi, Nicholas Black, John P. Farrant, Adam Pailing, Christopher Orsborne, Simon G. Williams, Erik B. Schelbert, Susanna Dodd, Richard Williams, Niels Peek, Matthias Schmitt, Theresa McDonagh, Christopher A. Miller

**Affiliations:** ^1^ Division of Cardiovascular Sciences, School of Medical Sciences, Faculty of Biology, Medicine and Health, Manchester Academic Health Science Centre University of Manchester Oxford Road Manchester M13 9PL UK; ^2^ Manchester University NHS Foundation Trust Manchester UK; ^3^ Minneapolis Heart Institute United Hospital Saint Paul MN USA; ^4^ Minneapolis Heart Institute Abbott Northwestern Hospital Minneapolis MN USA; ^5^ Department of Health Data Science University of Liverpool Liverpool UK; ^6^ Division of Informatics, Imaging and Data Science, School of Health Sciences, Faculty of Biology, Medicine and Health, Manchester Academic Health Science Centre University of Manchester Manchester UK; ^7^ NIHR Applied Research Collaboration Greater Manchester, Faculty of Biology, Medicine and Health, Manchester Academic Health Science Centre University of Manchester Manchester UK; ^8^ King's College Hospital London UK; ^9^ Wellcome Centre for Cell‐Matrix Research, Division of Cell Matrix Biology and Regenerative Medicine, School of Biology, Faculty of Biology, Medicine and Health, Manchester Academic Health Science Centre University of Manchester Manchester UK

**Keywords:** Heart failure, Hospitalization for heart failure, NHS Digital

## Abstract

**Aims:**

Population‐wide, person‐level, linked electronic health record data are increasingly used to estimate epidemiology, guide resource allocation, and identify events in clinical trials. The accuracy of data from NHS Digital (now part of NHS England) for identifying hospitalization for heart failure (HHF), a key HF standard, is not clear. This study aimed to evaluate the accuracy of NHS Digital data for identifying HHF.

**Methods and results:**

Patients experiencing at least one HHF, as determined by NHS Digital data, and age‐ and sex‐matched patients not experiencing HHF, were identified from a prospective cohort study and underwent expert adjudication. Three code sets commonly used to identify HHF were applied to the data and compared with expert adjudication (I50: International Classification of Diseases‐10 codes beginning I50; OIS: Clinical Commissioning Groups Outcomes Indicator Set; and NICOR: National Institute for Cardiovascular Outcomes Research, used as the basis for the National Heart Failure Audit in England and Wales). Five hundred four patients underwent expert adjudication, of which 10 (2%) were adjudicated to have experienced HHF. Specificity was high across all three code sets in the first diagnosis position {I50: 96.2% [95% confidence interval (CI) 94.1–97.7%]; NICOR: 93.3% [CI 90.8–95.4%]; OIS: 95.6% [CI 93.3–97.2%]} but decreased substantially as the number of diagnosis positions expanded. Sensitivity [40.0% (CI 12.2–73.8%)] and positive predictive value (PPV) [highest with I50: 17.4% (CI 8.1–33.6%)] were low in the first diagnosis position for all coding sets. PPV was higher for the National Heart Failure Audit criteria, albeit modestly [36.4% (CI 16.6–62.2%)].

**Conclusions:**

NHS Digital data were not able to accurately identify HHF and should not be used in isolation for this purpose.

## Introduction

The COVID‐19 pandemic highlighted the utility of population‐wide, person‐level, linked electronic health record data in the United Kingdom.^1^ Inclusion of people and events on a long‐term, nationwide level provides unparalleled opportunities to determine associations between risk factors and health outcomes whilst maximizing generalizability and precision of findings. ‘Data‐enabled’ trials, which utilize routine electronic health record data to identify potential participants, collect outcomes and potentially monitor safety, and ‘data‐enabled’ cohort studies, which supplement manually collected data with electronic health record data, offer considerable efficiencies.^2^

NHS Digital (now part of NHS England) provides data regarding hospital ‘episodes’, including hospital admissions, for all National Health Service (NHS)‐funded hospitals in England.^3^ Almost all non‐elective hospital admissions in England occur within these hospitals. International Classification of Diseases (ICD)‐10 codes are used to classify health conditions. Up to 20 diagnoses are listed for each hospital admission, with the first considered the primary diagnosis for that admission. Conventionally, codes are assigned by administrative coding teams at individual hospitals, based on review of medical records.

However, when using electronic health record data, for example, for research and national audits, there is a lack of consensus regarding which ICD‐10 codes should be applied to identify many conditions. Furthermore, due to multimorbidity, which is increasingly common, it is unclear how many diagnosis positions (i.e. 1–20) is optimal to consider.^4^ Clearly, variability in how the data are used is likely to impact research and audit findings.^5^

The high prevalence, poor prognosis, and large economic cost of heart failure (HF) make it a global health priority.^6^ Hospitalization for heart failure (HHF), in particular, portends an extremely poor prognosis, has a major adverse impact on quality of life, and accounts for the majority of costs associated with HF.^7^ Reflecting these factors, most HF studies include HHF in their primary outcome.

This study aimed to assess the accuracy of NHS Digital data for identification of HHF. Specifically, three ICD‐10 code sets that are commonly used for identifying HHF from NHS Digital data were compared with expert adjudication.

## Methods

### Study population

Participants were identified from a prospective longitudinal cohort study (NCT02326324), which has been described previously.^8^ Briefly, consecutive patients undergoing clinically indicated cardiovascular magnetic resonance imaging (CMR) at Manchester University NHS Foundation Trust, UK, between 1 April 2016 and 31 May 2018 were prospectively recruited. Exclusion criteria included previous HHF or a diagnosis of any of the following: amyloidosis, complex congenital heart disease, Fabry disease, hypertrophic cardiomyopathy, iron overload, myocarditis, and stress‐induced cardiomyopathy. The study was approved by the North West‐Greater Manchester West Research Ethics Committee, and all participants provided written informed consent.

The current study (‘adjudication cohort’) included patients being managed at the regional cardiac centre who experienced at least one HHF, as determined by the presence of any ICD‐10 code that forms part of the three HF code sets described below, in any of the 20 diagnosis positions (also see below), from data obtained from NHS Digital. A propensity score‐matched (based on age and sex) group of patients being managed at the regional cardiac centre, but without an ICD‐10 code from the three HF code sets in any diagnosis position, was also included.

### Outcome

Study outcome was HHF occurring after CMR. In the event of a participant having more than one HHF episode, only the first occurring episode was selected. The follow‐up period was from the beginning of recruitment (1 June 2016) until 19 August 2020.

### Identification of hospitalization for heart failure from NHS Digital data

Hospital Episode Statistics for Admitted Patient Care records, which includes NHS hospital encounters for almost the entire population of England, were obtained from NHS Digital (https://digital.nhs.uk) and used to identify hospital admissions. HHF was identified using three commonly used ICD‐10 code sets (descriptors for individual ICD‐10 codes are included in Supporting Information, *Table* *S1*):

1. I50: ICD‐10 codes beginning I50. This definition of HF is widely used.^9^, ^10^, ^11^
2. OIS: The Clinical Commissioning Groups Outcomes Indicator Set (OIS) was used by English healthcare commissioners to evaluate quality of health services and associated health outcomes. The OIS definition of HF comprises ICD‐10 codes I11.0, I13.0, I13.2, I25.5, I50.0, I50.1, and I50.9.^12^
3. NICOR: The UK National Institute for Cardiovascular Outcomes Research (NICOR) definition of HF, which comprises ICD‐10 codes I11.0, I25.5, I42.0, I42.9, I50.0, I50.1, and I50.9.

The impact of applying these code sets in different diagnosis positions was evaluated: specifically, (i) first (‘primary’) diagnosis position only, (ii) Diagnosis Positions 1 or 2, (iii) Diagnosis Positions 1–3, (iv) Diagnosis Positions 1–5, and (v) Diagnosis Positions 1–20. In addition, the impact of using the National Heart Failure Audit (for England and Wales) criteria to identify HHF was evaluated.^13^ The National Heart Failure Audit considers the NICOR code set in the first diagnosis position only, excludes patients admitted to hospital for elective procedures, and requires evidence of cardiac dysfunction on echocardiography (unless the patient has atrial fibrillation).

### Expert adjudication of hospitalization for heart failure

All HHF identified via NHS Digital data were adjudicated by expert review of medical records. In addition, medical records of matched participants with no HHF identified via NHS Digital data also underwent expert review. Adjudication was performed in multiple stages by a team composed of an undergraduate medical student, a junior medical trainee, a cardiology specialist registrar, and a consultant cardiologist with specialist expertise in HF. Adjudication was performed blinded to the ICD‐10 codes. The definition of adjudicated HHF was similar to Kalogeropoulos *et al*.^14^ and required physician documentation of HHF and (1) documented HF symptoms (e.g. shortness of breath, fatigue, orthopnoea, and paroxysmal nocturnal dyspnoea) and (2) supporting clinical findings (e.g. pulmonary oedema on radiography), or therapy for HF with diuretics. Medical records of participants identified as having HHF in the first diagnosis position from NHS Digital data, but which were deemed not to be HHF upon expert adjudication (i.e. false positives), were explored to identify the correct hospital admission diagnosis.

### Statistical analysis

Baseline characteristics and HHF rates were compared descriptively. Sensitivity, specificity, positive predictive value (PPV), and negative predictive value (NPV) for each HF code set according to diagnosis positions were calculated. PPVs and NPVs were adjusted for HHF prevalence.

## Results

### Participant characteristics

**Figure 1:**
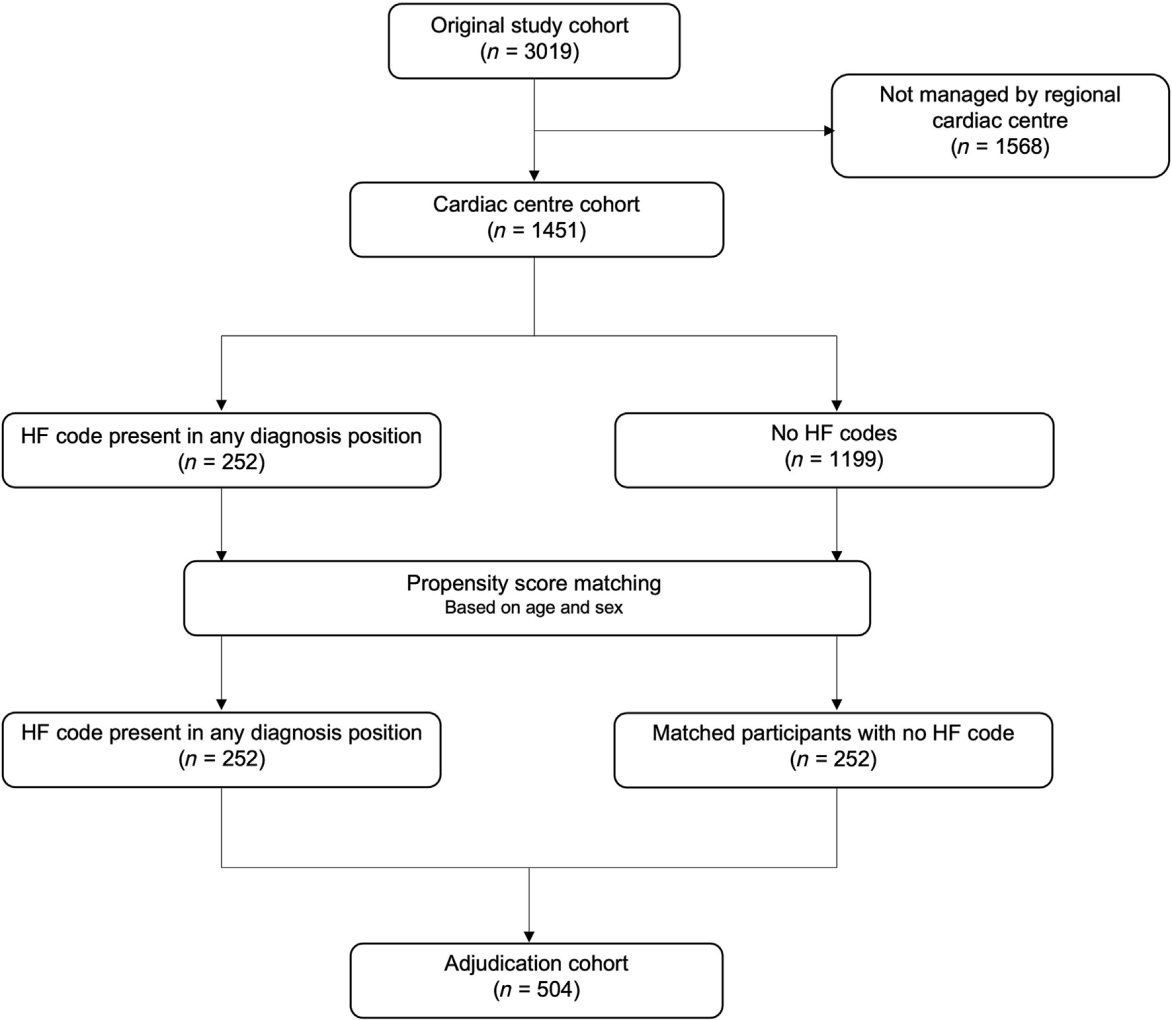
STROBE diagram. Heart failure (HF) International Classification of Diseases‐10 codes include I11.0, I13.0, I13.2, I25.5, I42.0, I42.9, I50.0, I50.1, and I50.9.

The overall study cohort comprised 3019 participants, of which 1449 were managed at the regional cardiac centre (*Figure* *1*). Outcome data, obtained from NHS Digital, were available for all participants. Median follow‐up duration was 1118 (interquartile range 950–1324) days. No participants were lost to follow‐up. The adjudication cohort comprised 504 participants, including 252 patients with an HF ICD‐10 code and 252 age‐ and sex‐matched participants with no HF ICD‐10 code. Baseline characteristics are presented in *Table* *1*. Characteristics of the overall cohort and the group managed at the regional cardiac centre were very similar. Participants in the adjudication cohort were older [adjudication cohort: 65 (54–72) vs. overall cohort: 58 (46–68)], diabetes was more common (17.3% vs. 14.2%), and N‐terminal prohormone of brain natriuretic peptide was higher [234.6 (86.4–774.8) vs. 127.1 (53.9–412.3) pg/mL], in keeping with patients being selected for the adjudication cohort on the basis of having an HF ICD‐10 code.

**Table 1:** Baseline characteristics

	Overall study cohort (*n* = 3019)	Cardiac centre cohort (*n* = 1451)	Adjudication cohort (*n* = 504)
Demographics			
Age	58 [46–68]	58 [46–68]	65 [54–72]
Male	1907 (63.2%)	932 (64.2%)	364 (72.2%)
Ethnicity			
White	2517 (83.4%)	1196 (82.4%)	430 (85.3%)
Asian	133 (4.4%)	72 (5%)	22 (4.4%)
Black	81 (2.7%)	48 (3.3%)	8 (1.6%)
Other	48 (1.6%)	31 (2.1%)	6 (1.3%)
Not declared	240 (7.9%)	87 (6%)	31 (6.2%)
BMI (kg/m^2^)	27.8 [24.5–31.7]	27.7 [24.3–31.8]	27.8 [24.7–31.9]
Medical history			
Percutaneous coronary intervention	399 (13.2%)	264 (18.2%)	107 (21.2%)
Coronary artery bypass graft	181 (6.0%)	101 (7%)	51 (10.1%)
Stroke or transient ischaemic attack	206 (6.8%)	95 (6.5%)	45 (8.9%)
Peripheral vascular disease	117 (3.9%)	53 (3.7%)	28 (5.6%)
Diabetes	429 (14.2%)	198 (13.6%)	87 (17.3%)
Hypertension	1337 (44.3%)	658 (45.3%)	275 (54.6%)
Hypercholesterolaemia	1302 (43.1%)	653 (45%)	271 (53.8%)
Chronic obstructive pulmonary disease	180 (6.0%)	76 (5.2%)	38 (7.5%)
Atrial fibrillation	417 (13.8%)	202 (13.9%)	94 (18.7%)
Current or past smoker	1465 (48.5%)	699 (48.2%)	258 (51.2%)
Laboratory indices			
eGFR (mL/min)	83 [70–90]	83 [70–90]	81 [68–90]
NT‐proBNP (pg/mL)	127.1 [53.9–412.3]	121.2 [53.8–328.2]	234.6 [86.4–774.8]
Cardiac structure and function			
Left ventricular ejection fraction (%)	56.2 ± 12.2	57.6 ± 11.1	53.2 ± 12.8

BMI, body mass index; eGFR, estimated glomerular filtration rate; NT‐proBNP, N‐terminal prohormone of brain natriuretic peptide.

Data are median (interquartile range), *n* (%), or mean (SD). Cardiac centre cohort = patients being managed at the regional cardiac centre. Adjudication cohort = patients for whom hospitalization for heart failure was expertly adjudicated.

### Adjudicated hospitalization for heart failure

Upon expert adjudication, 10 patients in the adjudication cohort were determined to have experienced HHF (2%). All 10 were patients with a HF ICD‐10 code. No matched patients (i.e. with no HF ICD‐10 code) were adjudicated to have HHF.

### Frequency of hospitalization for heart failure according to International Classification of Diseases‐10 codes and code sets

The frequency of HF ICD‐10 codes across diagnosis positions is displayed in Supporting Information, *Table* *S1*. Codes beginning I50 were the most frequent; for example, I50 codes accounted for 23 of 37 (62%) patients with an HF code in the first diagnosis position. ICD‐10 codes I11.0, I13.0, and I13.2 were not used in any patient.

When applying the three ICD‐10 code sets, observed rates of HHF using NHS Digital outcome data differed substantially compared with expert adjudication (*Table* *2* and Supporting Information, *Tables* *S2* and *S3*). The observed difference was less after accounting for patients admitted for elective procedures (in keeping with the National Heart Failure Audit) but remained substantial. The disparity between NHS Digital and adjudication worsened as the number of diagnosis positions expanded.

**Table 2:** Hospitalization for heart failure according to International Classification of Diseases‐10 code set and diagnosis position

Code set	Diagnosis Position 1				Diagnosis Positions 1–3				Diagnosis Positions 1–20			
	HHF		No HHF		HHF		No HHF		HHF		No HHF	
	NHS Digital	Adjudicated	NHS Digital	Adjudicated	NHS Digital	Adjudicated	NHS Digital	Adjudicated	NHS Digital	Adjudicated	NHS Digital	Adjudicated
I50	23	4	481	475	133	8	371	369	220	10	284	284
NICOR	37	4	467	461	161	10	343	343	252	10	252	252
OIS	26	4	378	472	136	9	368	367	224	10	280	280
NHFA^a^	11	4	493	487								

HHF, hospitalization for heart failure; NHFA, National Heart Failure Audit in England and Wales; NHS, National Health Service; NICOR, the National Institute for Cardiovascular Outcomes Research; OIS, the Clinical Commissioning Groups Outcomes Indicator Set.

I50 code set includes all International Classification of Diseases‐10 codes beginning I50. There was a total of 10 adjudicated HHF episodes. See Supporting Information, *Table* *S2* for 2 × 2 Tables, and Supporting Information, *Table* *S3* for Diagnosis Positions 1–2 and 1–5.

^a^ The NHFA uses the NICOR code set to identify HHF but excludes patients admitted for elective cardiac procedures.

### Sensitivity, specificity, and predictive value of code sets

Specificity was high for all three code sets in the first diagnosis position {I50: 96.2% [95% confidence interval (CI) 94.1–97.7%]; NICOR: 93.3% [CI 90.8–95.4%]; OIS: 95.6% [CI 93.3–97.2%]} but decreased substantially as the number of diagnosis positions expanded (*Table* *3* and Supporting Information, *Table* *S4*). NPV was high across all code sets in all diagnosis positions. Conversely, sensitivity was low in the first diagnosis position [40.0% (CI 12.2–73.8%) for each coding set] and improved upon expansion of diagnosis positions. PPVs were low for all coding sets in all diagnosis positions, with the I50 code set in the first diagnosis position demonstrating the highest PPV [17.4% (CI 8.1–33.6%)]. PPV was higher for the National Heart Failure Audit criteria, albeit modestly [36.4% (CI 16.6–62.2%)].

**Table 3:** Sensitivity, specificity, and predictive values of heart failure International Classification of Diseases‐10 code sets according to diagnosis positions

Code set	Diagnosis Position 1				Diagnosis Positions 1–3				Diagnosis Positions 1–20			
	Sensitivity (95% CI)	Specificity (95% CI)	PPV (95% CI)	NPV (95% CI)	Sensitivity (95% CI)	Specificity (95% CI)	PPV (95% CI)	NPV (95% CI)	Sensitivity (95% CI)	Specificity (95% CI)	PPV (95% CI)	NPV (95% CI)
I50	40% (12.2–73.8)	96.2% (94.1–97.7)	17.4% (8.1–33.6)	98.8% (98–99.2)	80% (44.4–97.5)	74.7% (70.6–78.5)	6% (4.3–8.3)	99.5% (98.2–99.9)	100% (69.2–100)	57.5% (53–61.9)	4.6% (4.1–5)	100% (98.7–100)
NICOR	40% (12.2–73.8)	93.3% (90.8–95.4)	10.8% (5–21.7)	98.7% (97.9–99.2)	100% (69.2–100)	69.43% (65.2–73.5)	6.2% (5.5–7)	100% (98.9–100)	100% (69.2–100)	51% (46.5–55.5)	4% (3.6–4.3)	100% (98.6–100)
OIS	40% (12.2–73.8)	95.6% (93.3–97.2)	15.4% (7.1–30.1)	98.7% (97.9–99.2)	90% (55.5–99.8)	74.3% (70.2–78.1)	6.6% (5.2–8.4)	99.7% (98.3–100)	100% (69.2–100)	56.7% (52.2–61.1)	4.5% (4.1–4.9)	100% (98.7–100)
NHFA^a^	40% (12.2–73.8)	98.6% (97.1–99.4)	36.4% (16.6–62.2)	98.8% (98–99.3)								

CI, confidence interval; NPV, negative predictive value; PPV, positive predictive value.

See *Table* *2* for other abbreviations and description, and Supporting Information, *Table* *S4* for Diagnosis Positions 1–2 and 1–5.

^a^ The NHFA uses the NICOR code set to identify HHF but excludes patients admitted for elective cardiac procedures.

### Hospitalization for heart failure false positives

Thirty‐three patients identified as having HHF via an HF ICD‐10 in the first diagnosis position were deemed not to have HHF on expert adjudication (false positives). The true reasons for the hospital admissions in these patients are given in Supporting Information, *Table* *S5*. Elective admission for a procedure was the most common reason (26 patients, 79%), of which cardiac device implantation or revision was the most frequent (21 patients, 64%).

## Discussion

This study is the first to expertly adjudicate HHF outcomes obtained from NHS Digital. The principal findings are that NHS Digital data were not able to accurately identify HHF. Specifically, ICD‐10 code sets commonly used to identify HHF from NHS Digital data were found to have high specificity but low sensitivity and PPV.

Outcomes in phase III HF trials are typically adjudicated by independent clinical events committees, the aims of which are to provide consistent, unbiased, and blinded evaluation of suspected endpoints, in an effort to maximize treatment effect estimate precision and, thus, increase the likelihood of identifying efficacy if it is indeed present.^15^ However, such committees are expensive and time consuming, and adjudication accuracy is dependent on the information provided by the site clinical teams and the population being studied. The nature of events, particularly reason for hospitalization, can be difficult to determine. Outcomes in cohort studies are often not adjudicated, instead traditionally relying on research teams identifying endpoints via hospital and primary care record review, or by contacting patients, which is time consuming and prone to being incomplete.

NHS Digital data include all NHS hospital encounters for almost the entire population of England (more than 54 million people). Similar health informatics systems are available in Scotland (Scottish National Data Safe Haven) and Wales (SAIL Databank). Such resources are highly attractive for outcome data collection for trials and cohort studies. Once the appropriate information governance is in place, events can potentially be identified quickly, cheaply, and at scale, either at a single timepoint or at regular intervals. The drive by governmental and charitable research funders for more pragmatic, efficient, and thus less expensive research has led to an increasing number of ‘data‐driven’ trials and cohort studies that utilize health informatics systems for identifying endpoints.^2^ However, for many conditions, it is not clear how to optimally identify events of interest from the data held, resulting in marked variability in how data are used, which potentially impacts the accuracy of trial results and prognostic model performance.

As demonstrated by the code sets included in the current study, there is no consensus regarding which ICD‐10 codes to use to identify HHF, or which diagnosis positions to apply the codes to. There is overlap between NICOR and OIS code sets (I11.0, I25.5, I50.0, I50.1, and I50.9), but also differences (OIS includes I13.0 and I13.2 but NICOR does not; NICOR includes I42.0 and I42.9 but OIS does not). All but one (I25.5, Ischaemic cardiomyopathy) of the seven codes included in the OIS code set include the term ‘HF’, whereas three of the seven codes included in the NICOR set do not include the term ‘HF’ (I25.5, Ischaemic cardiomyopathy; I42.0, Dilated cardiomyopathy; and I42.9, Cardiomyopathy, unspecified). It is not clear how such code sets have evolved. Dilated cardiomyopathy, for example, is a substrate for HF but is not HF as such (see Supporting Information, *Table* *S6* for a comparison of characteristics of patients with first diagnosis position I50 and I42.0 codes). As is evident from *Table* *2* and Supporting Information, *Table* *S1*, I50 codes are the codes that are being most commonly used by hospital administrative coding teams for HF. I11.0, I13.0, and I13.2 are not being used for HF.

The use of the first diagnosis position resulted in high specificity for the three code sets. However, sensitivity for all code sets was 40%, improving only upon expansion of diagnosis positions, at the expense of specificity. PPVs were universally low; indeed, the NICOR code set provided the lowest PPV and resulted in the highest number of false positive HHF episodes (first diagnosis position only), of which the majority were admissions for elective cardiac procedures (73%). The National Heart Failure Audit, responsible for reporting HF outcomes in England and Wales, uses the NICOR code set in the first diagnosis position but excludes patients admitted for elective cardiac procedures and requires patients to have evidence of cardiac dysfunction on cardiac imaging, or have a concomitant diagnosis of atrial fibrillation. Whilst this datum is available to NICOR via other data sets, it is not readily available via NHS Digital to other research teams. No specific ICD‐10 codes exist for elective cardiac procedures. Importantly, even after accounting for elective procedures in the current study, sensitivity and PPV remained low, suggesting that reported HHF in England and Wales is potentially inaccurate.

Inaccurate coding of hospital admissions by administrative, non‐medically trained, coding teams is likely a key factor underlying the findings. Increasingly complex hospital admissions, involving management of multiple conditions, often make it difficult to determine cause of admission accurately. Furthermore, coding policies, training pathways, and guidance vary between hospitals. The transition to electronic health records, where reasons for admission (and their associated ICD‐10 codes) are inputted directly by medical staff, should result in greater accuracy. Nevertheless, variability in how ICD‐10 codes are used to identify conditions will continue to confound data outputs. National and global consensus is required.

The findings of the current study clearly have important implications for research and audit using data from NHS Digital, and hence, for estimating HF disease burden in England and Wales, and, crucially, resource allocation. It is unlikely that HF is unique, and similar inaccuracies are expected for other conditions.^16^, ^17^, ^18^ Validation studies of outcome data obtained from NHS Digital are required across the spectrum of disease, in keeping with similar studies of Scandinavian patient registries.^19^

A limitation of the present investigation is only including patients being managed at the regional cardiac centre. This was done to facilitate accurate adjudication; indeed, it highlights the need for a resource, such as NHS Digital. Nevertheless, baseline characteristics were similar to the wider cohort. The rate of elective cardiac procedures could potentially be expected to be higher in a regional cardiac centre population; however, these were predominantly cardiac device implantation, which is carried out routinely in district general hospitals. A further limitation is the inclusion of elective hospital admissions in the data obtained from NHS Digital. Whilst NHS Digital does provide means of filtering for unplanned admissions only, it is unclear how accurate this information is, and excluding them resulted in only a modest improvement in sensitivity and PPV.

In conclusion, NHS Digital data do not accurately identify HHF. The findings have important implications for the use of NHS Digital data for cardiovascular audit and research and, potentially, for resource allocation.

## Conflict of interest

C.A.M., Advanced Fellowship, NIHR301338 is funded by the National Institute for Health and Care Research (NIHR) and acknowledges support from the University of Manchester British Heart Foundation Accelerator Award (AA/18/4/34221) and the NIHR Manchester Biomedical Research Centre (NIHR203308); C.A.M. has participated on advisory boards/consulted for AstraZeneca, Boehringer Ingelheim and Lilly Alliance, Novartis, and PureTech Health, serves as an advisor for HAYA Therapeutics, and has received speaker fees from AstraZeneca, Boehringer Ingelheim, and Novo Nordisk, conference attendance support from AstraZeneca, and research support from Amicus Therapeutics, AstraZeneca, Guerbet Laboratories Limited, Roche, and Univar Solutions B.V.

## Funding

The cohort study was funded in part by the UK National Institute for Health and Care Research (NIHR, CS‐2015‐15‐003) and supported by a research grant from Guerbet Laboratories Limited. The work was also supported in part by a British Heart Foundation Accelerator Award to The University of Manchester (BHF, AA/18/4/34221).

## Supporting information


**Table S1.** ICD‐10 Code definitions and frequencies according to diagnosis position and adjudication.
**Table S2.** HHF according to ICD‐10 code set and diagnosis position (2x2 Tables).
**Table S3.** HHF according to ICD‐10 code set and diagnosis position.
**Table S4.** Sensitivity, specificity, PPV, NPV and accuracy of HF ICD‐10 code sets according to diagnosis positions.
**Table S5.** True reason for hospital admission for false positives identified in the first diagnosis position.
**Table S6.** Baseline characteristics of patients with first diagnosis position I50 code versus I42.0 Code (Dilated Cardiomyopathy).
