# Supplementary material for: Identification of heart failure hospitalization from NHS Digital data: comparison with expert adjudication
Source: ESC Heart Fail. 2024 Jan 17;11(2):1022–9. doi: 10.1002/ehf2.14669 (PMC10966211; doi:10.1002/ehf2.14669)
Supplement: Supplementary file 1 — Table S1. ICD‐10 Code definitions and frequencies according to diagnosis position and adjudication. Table S2. HHF according to ICD‐10 code set and diagnosis position (2x2 Tables). Table S3. HHF according to ICD‐10 code set and diagnosis position. Table S4. Sensitivity, specificity, PPV, NPV and accuracy of HF ICD‐10 code sets according to diagnosis positions. Table S5. True reason for hospital admission for false positives identified in the first diagnosis position. Table S6. Baseline characteristics of patients with first diagnosis position I50 code versus I42.0 Code (Dilated Cardiomyopathy). [file EHF2-11-1022-s001.docx]

**Identification of heart failure hospitalisation from NHS Digital data: comparison with expert adjudication**

**Supplementary appendix**

**Table of Contents**

[Supplemental Tables 3](#_Toc142909533)

[Supplemental Table 1. ICD-10 Code definitions and frequencies according to diagnosis position and adjudication 4](#_Toc142909534)

[Supplemental Table 2. HHF according to ICD-10 code set and diagnosis position (2x2 Tables) 5](#_Toc142909535)

[Supplemental Table 3. HHF according to ICD-10 code set and diagnosis position 6](#_Toc142909536)

[Supplemental Table 4. Sensitivity, specificity, PPV, NPV and accuracy of HF ICD-10 code sets according to diagnosis positions 7](#_Toc142909537)

[Supplemental Table 5. True reason for hospital admission for false positives identified in the first diagnosis position 8](#_Toc142909538)

[Supplemental Table 6. Baseline characteristics of patients with first diagnosis position I50 code versus I42·0 Code (Dilated Cardiomyopathy) 9](#_Toc142909539)

# Supplemental Tables

## Supplemental Table 1. ICD-10 Code definitions and frequencies according to diagnosis position and adjudication

| **ICD-10 Code** | **ICD- 10 Code Definition** | **Diagnosis Position 1** | **Diagnosis Positions 1-2** | **Diagnosis Positions 1-3** | **Diagnosis Positions 1-5** | **Diagnosis Positions 1-20** | **Adjudicated hospitalisation for heart failure** |
| --- | --- | --- | --- | --- | --- | --- | --- |
| I11·0 | Hypertensive heart disease with (congestive) heart failure | 0 | 0 | 0 | 0 | 0 | 0 |
| I13·0 | Hypertensive heart and renal disease with (congestive) heart failure | 0 | 0 | 0 | 0 | 0 | 0 |
| I13·2 | Hypertensive heart and renal disease with both (congestive) heart failure and renal failure | 0 | 0 | 0 | 0 | 0 | 0 |
| I25·5 | Ischaemic cardiomyopathy | 3 | 7 | 12 | 18 | 20 | 1 |
| I42·0 | Dilated cardiomyopathy | 11 | 21 | 25 | 29 | 33 | 1 |
| I42·9 | Cardiomyopathy, unspecified | 0 | 8 | 15 | 25 | 32 | 0 |
| I50·0 | Congestive Heart Failure | 7 | 19 | 24 | 31 | 39 | 5 |
| I50·1 | Left ventricular failure | 14 | 56 | 98 | 126 | 151 | 1 |
| I50·9 | Heart failure, unspecified | 2 | 5 | 11 | 17 | 30 | 2 |

Values represent patient frequencies of ICD-10 codes, rather than specific HHF episodes. Each HHF episode may include multiple heart failure ICD-10 codes in different diagnosis positions. Only the first occurring HHF episode for patients was included.

## Supplemental Table 2. HHF according to ICD-10 code set and diagnosis position (2x2 Tables)

A

| **I50 Code Set** | | **NHS Digital** | | | | | |
| --- | --- | --- | --- | --- | --- | --- | --- |
|  |  | Diagnosis Position 1 | | Diagnosis Positions 1-3 | | Diagnosis Positions 1-20 | |
|  |  | HHF | No HHF | HHF | No HHF | HHF | No HHF |
| **Adjudication (True Disease)** | HHF | 4 | 6 | 8 | 2 | 10 | 0 |
|  | No HHF | 19 | 475 | 125 | 369 | 210 | 284 |

B

| **NICOR Code Set** | | **NHS Digital** | | | | | |
| --- | --- | --- | --- | --- | --- | --- | --- |
|  |  | Diagnosis Position 1 | | Diagnosis Positions 1-3 | | Diagnosis Positions 1-20 | |
|  |  | HHF | No HHF | HHF | No HHF | HHF | No HHF |
| **Adjudication (True Disease)** | HHF | 4 | 6 | 10 | 0 | 10 | 0 |
|  | No HHF | 33 | 461 | 151 | 343 | 242 | 252 |

C

| **OIS Code Set** | | **NHS Digital** | | | | | |
| --- | --- | --- | --- | --- | --- | --- | --- |
|  |  | Diagnosis Position 1 | | Diagnosis Positions 1-3 | | Diagnosis Positions 1-20 | |
|  |  | HHF | No HHF | HHF | No HHF | HHF | No HHF |
| **Adjudication (True Disease)** | HHF | 4 | 6 | 9 | 1 | 10 | 0 |
|  | No HHF | 22 | 472 | 127 | 367 | 214 | 280 |

D

| **NHFA*** | | **NHS Digital** | |
| --- | --- | --- | --- |
|  |  | Diagnosis Position 1 | |
|  |  | HHF | No HHF |
| **Adjudication (True Disease)** | HHF | 4 | 6 |
|  | No HHF | 7 | 487 |

OIS indicates The Clinical Commissioning Groups Outcomes Indicator Set; NHFA, National Heart Failure Audit in England and Wales; NICOR, The National Institute for Cardiovascular Outcomes Research. I50 code set includes all ICD-10 codes beginning I50. There was a total of 10 adjudicated HHF episodes. *The NHFA uses the NICOR code set to identify HHF, but excludes patients admitted for elective cardiac procedures.

## Supplemental Table 3. HHF according to ICD-10 code set and diagnosis position

| **Code Set** | **Diagnosis Positions 1-2** | | | | **Diagnosis Positions 1-5** | | | |
| --- | --- | --- | --- | --- | --- | --- | --- | --- |
|  | **HHF** | | **No HHF** | | **HHF** | | **No HHF** | |
|  | NHS Digital | Adjudicated | NHS Digital | Adjudicated | NHS Digital | Adjudicated | NHS Digital | Adjudicated |
| I50 | 80 | 6 | 424 | 420 | 174 | 10 | 330 | 330 |
| NICOR | 103 | 7 | 401 | 398 | 204 | 10 | 300 | 300 |
| OIS | 83 | 6 | 421 | 417 | 176 | 10 | 328 | 328 |

OIS indicates The Clinical Commissioning Groups Outcomes Indicator Set; NHFA, National Heart Failure Audit in England and Wales; NICOR, The National Institute for Cardiovascular Outcomes Research. I50 code set includes all ICD-10 codes beginning I50. There was a total of 10 adjudicated HHF episodes. See Table 2 for Diagnosis Positions 1, 1-3, and 1-20.

## Supplemental Table 4. Sensitivity, specificity, PPV, NPV and accuracy of HF ICD-10 code sets according to diagnosis positions

| **Code**  **Set** | **Diagnosis Positions 1-2** | | | | **Diagnosis Positions 1-5** | | | |
| --- | --- | --- | --- | --- | --- | --- | --- | --- |
|  | Sensitivity  (95% CI) | Specificity  (95% CI) | PPV  (95% CI) | NPV  (95% CI) | Sensitivity  (95% CI) | Specificity  (95% CI) | PPV  (95% CI) | NPV  (95% CI) |
| I50 | 60%  (26·2-87·8) | 85%  (81·6-88·1) | 7·5%  (4·5-12·3) | 99·1%  (98-99·6) | 100%  (69·2-100) | 66·8%  (62·5-70·9) | 5·8%  (5·1-6·5) | 100%  (98·9-100) |
| NICOR | 70%  (34·8-93·3) | 80·6%  (76·8-84) | 6·8%  (4·5-10·2) | 99%  (98·1-99·7) | 100%  (69·2-100) | 60·7%  (56·3-65·1) | 4·9%  (4·4-5·4) | 100%  (98·8-100) |
| OIS | 60%  (26·2-87·8) | 84·4%  (80·9-87·5) | 7·2%  (4·3-11·9) | 99·1%  (98-99·6) | 100%  (69·2-100) | 66·4%  (62-70.6) | 5·7%  (5·1-6·4) | 100%  (98·9-100) |

See previous table for abbreviations and description, and Table 3 for Diagnosis Positions 1, 1-3, and 1-20.

## Supplemental Table 5. True reason for hospital admission for false positives identified in the first diagnosis position

| **ICD-10 Code in Diagnosis Position 1** | **True reason for hospital admission** |
| --- | --- |
| I501 | Elective cardiac device implantation |
| I501 | Elective cardiac device implantation |
| I501 | Elective cardiac device lead revision |
| I501 | Elective cardiac device implantation |
| I500 | Elective cardiac device lead revision |
| I501 | Elective cardiac device implantation |
| I500 | Anticoagulation appointment |
| I501 | Elective cardiac device implantation |
| I501 | Elective cardiac device implantation |
| I501 | Elective cardioversion |
| I500 | Gastritis |
| I501 | Elective cardiac device implantation |
| I501 | Elective cardiac device implantation |
| I501 | Myocarditis |
| I501 | Blood Transfusion |
| I509 | Side effects of chemotherapy |
| I501 | Elective cardiac device implantation |
| I420 | Elective cardiac catheterisation |
| I255 | Anticoagulation appointment |
| I420 | Elective cardiac device implantation |
| I420 | Elective cardiac device implantation |
| I255 | Elective cardiac catheterisation |
| I420 | Elective cardiac device implantation |
| I420 | Elective cardiac device implantation |
| I255 | Elective cardiac device implantation |
| I420 | Elective cardiac device implantation |
| I420 | Elective cardiac device implantation |
| I420 | Vasovagal episode |
| I420 | Elective cardiac device implantation |
| I420 | Elective cardiac device implantation |
| I420 | Elective cardiac device implantation |
| I500 | Syncope |
| I501 | Obesity Sleep Apnoea |

## Supplemental Table 6. Baseline characteristics of patients with first diagnosis position I50 code versus I42·0 Code (Dilated Cardiomyopathy)

|  | **I50 Code Set**  **(n = 23)** | **I42·0 Code (Dilated Cardiomyopathy)**  **(n = 11)** |
| --- | --- | --- |
| **Demographics** |  |  |
| Age | 63 [53-72] | 57 [44·5-64] |
| Male | 14 (60·9%) | 10 (90·9%) |
| Ethnicity |  |  |
| White | 19 (82·6%) | 11 (100%) |
| Asian | 2 (8·7 %) | 0 (0%) |
| Black | 0 (0%) | 0 (0%) |
| Other | 0 (0%) | 0 (0%) |
| Not declared | 2 (8·7 %) | 0 (0%) |
| BMI (kg/m2) | 31·1 [27·2-34·5] | 28·4 [23·9-30·5] |
| **Medical history** |  |  |
| Percutaneous coronary intervention | 6 (26·1%) | 2 (18·2%) |
| Coronary artery bypass graft | 2 (8·7%) | 0 (0%) |
| Stroke or transient ischaemic attack | 1 (4·3%) | 1 (9·1%) |
| Peripheral vascular disease | 1 (4·3%) | 0 (0%) |
| Diabetes | 6 (26·1%) | 0 (0%) |
| Hypertension | 10 (43·5%) | 2 (18·2%) |
| Hypercholesterolaemia | 12 (52·2%) | 2 (18·2%) |
| Chronic obstructive pulmonary disease | 3 (13%) | 1 (9·1%) |
| Atrial fibrillation | 6 (26·1%) | 1 (9·1%) |
| Current or past smoker | 8 (34·8%) | 8 (72·7%) |
| **Laboratory indices** |  |  |
| eGFR (mL/min) | 81 [61-88·5] | 84 [78-90] |
| NT-proBNP (pg/mL) | 659 [73·8-1258·3] | 254·85 [140·4-1943·8] |
| **Cardiac structure and function** |  |  |
| Left ventricular ejection fraction (%) | 42·2 ± 14·2 | 37·4 ± 14·4 |

Data are median (IQR), n (%), or mean (SD). BMI indicates body mass index; eGFR, estimated glomerular filtration rate; NT-proBNP, N-terminal prohormone of brain natriuretic peptide.
